# Estimation of skin microcirculatory hemoglobin oxygen saturation and red blood cell tissue fraction using a multispectral snapshot imaging system: a validation study

**DOI:** 10.1117/1.JBO.26.2.026002

**Published:** 2021-02-13

**Authors:** Maria Ewerlöf, E. Göran Salerud, Tomas Strömberg, Marcus Larsson

**Affiliations:** Linköping University, Department of Biomedical Engineering, Linköping, Sweden

**Keywords:** multispectral imaging, hemoglobin oxygen saturation, RBC tissue fraction, diffuse reflectance spectroscopy, Monte Carlo simulations, skin microcirculation

## Abstract

**Significance**: Hemoglobin oxygen saturation and red blood cell (RBC) tissue fraction are important parameters when assessing microvascular status. Functional information can be attained using temporally resolved measurements performed during stimulus–response protocols. Pointwise assessments can currently be conducted with probe-based systems. However, snapshot multispectral imaging (MSI) can be used for spatial–temporal measurements.

**Aim**: To validate if hemoglobin oxygen saturation and RBC tissue fraction can be quantified using a snapshot MSI system and an inverse Monte Carlo algorithm.

**Approach**: Skin tissue measurements from the MSI system were compared to those from a validated probe-based system during arterial and venous occlusion provocation on 24 subjects in the wavelength interval 450 to 650 nm, to evaluate a wide range of hemoglobin oxygen saturation and RBC tissue fraction levels.

**Results**: Arterial occlusion results show a mean linear regression $R^{2}=0.958$ for hemoglobin oxygen saturation. Comparing relative RBC tissue fraction during venous occlusion results in $R^{2}=0.925$. The MSI system shows larger dynamic changes than the reference system, which might be explained by a deeper sampling including more capacitance vessels.

**Conclusions**: The snapshot MSI system estimates hemoglobin oxygen saturation and RBC tissue fraction in skin microcirculation showing a high correlation ($R^{2}>0.9$ in most subjects) with those measured by the reference method.

## 1 Introduction

Tissue microcirculation involves the smallest vessels of the circulatory system: a network of capillaries that supply cells in the body with oxygen and nutrients and simultaneously remove waste products emanating from the cell breathing cycle. When delivery of blood or cellular uptake of oxygen is severely impaired, cell failure is soon to be found in the affected region. Anomalies in distribution at a local microcirculatory level can reveal early signs of sickness through parameters such as hemoglobin oxygen saturation level and red blood cell (RBC) tissue fraction.^1^ Baseline values may reveal important physiological information, but more often the circulatory system’s ability to adapt to natural or induced stimuli is evaluated in clinical settings. Stimulus–response protocols reveal the microcirculatory system’s ability to constrict or dilate vessels and thereby regulate the blood flow to adapt to periods of ischemia or heating provocation.^2^^–^^5^

Oxy- and deoxyhemoglobin, found in RBCs, are strong absorbers in the visible wavelength range in human tissue with unique spectral fingerprints. The amount of oxy- and deoxyhemoglobin may be estimated using diffuse reflectance spectroscopy (DRS) to study hemoglobin oxygen saturation and RBC tissue fraction. DRS uses a broadband light source to irradiate tissue. Photons that enter the tissue interact with structures and chromophores through scattering and absorption processes. Backscattered photons that are detected and resolved spectrally thus carry information about tissue composition. Depending on the geometry in a fiber optic probe or in an imaging setup, different photon transportation models need to be applied to untangle this information. Simplified homogeneous or layered tissue models combined with diffusion theory or Monte Carlo simulations can be used to decompose the measured DRS signal.^6^

Multispectral imaging (MSI) and hyperspectral imaging (HSI) are DRS-based methods providing 2D images containing spectral information about every pixel, creating a 3D dataset, i.e., a data cube. The acquisition of data cubes is commonly done either by sweeping through the wavelength dimension (spectral scanning) or the spatial dimensions (spatial scanning). Spatial scanning can be done by acquiring the complete spectrum for a single pixel (whiskbroom technology) or for a line of pixels (pushbroom technology), and then scanning through the area of interest. In spectral scanning, a 2D image is captured at each exposure, which involves scanning through several wavelengths.^7^ In an occlusion-release experiment, Bjorgan et al.^8^ used a pushbroom hyperspectral camera system on the volar forearm to estimate oxygen saturation levels at baseline and after 5 min of occlusion. They found a systematic difference between Monte Carlo simulations and the diffusion model in estimating oxygen saturation, emphasizing the need for an accurate model when analyzing data cubes.

MSI snapshot cameras capture the full data cube (both spatial and spectral information) in a single exposure allowing for temporal changes of the microcirculation to be monitored.^9^ The spectral information is often achieved by applying filters to individual detector elements or by dispersing the light spectrally over the detector, which limits both spectral and spatial dimensions of the data cube.^10^ The snapshot MSI detector used in this work has a Fabry–Perot filter array with 16 filters distributed in a recurring 4×4 mosaic pattern over the sensor pixels.^11^ Both Bauer et al.^12^ and Rubins et al.^13^ used the described snapshot MSI camera to capture data during occlusion-release tests. Rubins et al. measured the palm of the hand and fitted data from 10 wavelength bands to modeled data using a two-layered diffusion model where hemoglobin oxygen saturation, RBC tissue fraction, melanin concentration, and epidermal thickness varied.^13^^,^^14^ Bauer et al.^12^ captured data from the back of the hand and analyzed intensity data from three spectrally corrected wavelength bands using a modified Beer–Lambert expression.

The Monte Carlo method for light transport has been considered a gold standard that allows light transport in arbitrarily complex geometries to be modeled at the expense of computational time.^6^ Therefore, we have used it in our previous work^15^ describing an MSI snapshot camera estimating hemoglobin oxygen saturation and RBC tissue fraction in skin microcirculation. In this paper, we have advanced the method by changing the white normalization procedure as well as the hemoglobin absorption spectrum. Moreover, the method was applied to a cohort of 24 subjects undergoing standardized vascular occlusion tests with large dynamic changes in hemoglobin oxygen saturation (arterial occlusion) and RBC tissue fraction (venous occlusion). The work aimed at extensive *in-vivo* validation of the MSI system’s ability to quantify hemoglobin oxygen saturation and the relative RBC tissue fraction, comparing MSI data to data co-registered with a probe-based reference system. The reference system analyzes DRS data in the same spectral range as the MSI system.^16^^,^^17^

## 2 Materials and Methods

### 2.1 Equipment

A snapshot MSI camera (xiSpec MQ022HG-IM-SM4X4-VIS, XIMEA®, Germany) was used to capture spectral data of diffusively scattered light from skin tissue during vascular occlusion provocation. The camera has a CMOS sensor (2048×1088  pixels) overlaid with a Fabry–Perot interference filter array. The array contains 16 unique bandpass filters aligned to the pixel layout in a recurring 4×4 mosaic pattern. Each filter has a specified spectral response in the wavelength range 450 to 650 nm with at least one main peak in the interval 470 to 630 nm and with substantial spectral overlap between filters. The camera was equipped with a 16-mm lens (C Series VIS-NIR Lens, Edmund Optics Inc., Barrington).

The MSI camera was mounted side-by-side to a ring light (R130, Smart Vision Lights) with eight LED sources emitting light in the visible range (390 to 765 nm). The LED light spectrum was characterized using a spectrometer (AvaSpec-ULS2048L-RS-USB2, Avantes BV, Apeldoorn, The Netherlands) calibrated with a reference lamp (AvaLight-HAL-CAL-Mini, Avantes BV, Apeldoorn, The Netherlands). To avoid surface specular reflections from the illuminated tissue, a crossed polarizer/analyzer pair was used with the polarizer placed in front of the ring light source and the analyzer in front of the detecting MSI camera.

Data were collected and analyzed using MATLAB (MATLAB version 8.2.0, 2013b, computer software, The MathWorks Inc., Natick, Massachusetts). To reduce noise and amount of data, 16 consecutive snapshots, each with exposure time 20 ms, were taken with the camera and then summed. The resulting image was stored during recording at an effective framerate of 1.4 frames per second (fps).

A probe-based PF6000 EPOS instrument (Perimed AB, Järfälla, Sweden) was used as a reference. It assesses absolute values for hemoglobin oxygen saturation (%) and RBC tissue fraction (% tissue fraction) with a sampling rate of 1 Hz. The system is based on an inverse Monte Carlo algorithm for analyzing spatially resolved diffuse reflectance spectra captured at two source–detector separations (0.4 and 1.2 mm) in the wavelength interval 450 to 850 nm. The inverse algorithm make use of a three-layer skin tissue model that accounts for individual variations in blood amount, blood oxygen saturation, melanin amount, layer thickness, and tissue scattering.^16^ Phantom experiments have demonstrated that the EPOS system estimates blood oxygen saturation to within 5% (RMS deviation) and the absolute RBC tissue fraction to within 11% (RMS deviation).^18^ The EPOS system was equipped with an AvaLight-HAL-S (Avantes, Apeldoorn, The Netherlands) light source for 11 subjects and changed to an AvaLight-HAL-S-Mini for the last 13 subjects. Both light sources emit light in the range of 360 to 2500 nm. The blood pressure arm cuff used for vascular occlusion was controlled by the EPOS.

### 2.2 Measurement Protocols

To capture dynamic changes in hemoglobin oxygen saturation and RBC tissue fraction, two separate provocation protocols (arterial and venous occlusion) were performed on the volar forearm of healthy subjects. Left or right arm was chosen randomly. Both protocols were performed on the same arm, starting with arterial occlusion. A resting period of at least 45 min was given after completion of the arterial occlusion protocol to ensure minimal influence from the earlier provocation.

The subject acclimatized to the room in a seated position for at least 15 min before the first protocol started. The arm rested comfortably on a stable surface where a vacuum pillow (Germa Protec, AB Germa, Kristianstad, Sweden) provided support and fixation throughout the measurements. To get a spatial reference, nine ink dots were placed on the volar forearm, which marked a square with four 2×2  cm regions of interest (ROI) (4×4  cm in total). The MSI camera was positioned 30 cm above the arm surface, with the dots within the field of view. Using double-adhesive tape, the EPOS probe was fixed to the volar forearm close to but outside the ROI, distally in relation to the marked area.

The blood pressure cuff was placed around the upper arm of the subject before starting the measurements. After 5 min of baseline measurements, the cuff was rapidly inflated to a pressure of 250 mmHg (arterial occlusion) or 45 mmHg (venous occlusion), respectively. The cuff upheld a constant pressure for 5 min and was then deflated. Measurements continued for 10 min after release to capture recovery after provocation.

### 2.3 Study Subjects

This study included 24 healthy persons (11 women and 13 men), aged 18 to 40 years with Caucasian skin, classified as type I to III (self-valuated from Fitzpatrick skin typing test^19^). Exclusion criteria were known skin conditions or circulatory diseases, use of medication affecting the circulatory system and smoking. Participants were asked to refrain from extensive physical activity for 24 h and coffee for 4 h prior to measurements. Written informed consent was obtained from all participants. The study was approved by the Regional Ethical Review Board in Linköping (D. No. 2015/392-31).

### 2.4 Data Collection

The MSI camera acquired data at 1.4 fps throughout the length of each protocol. The EPOS system measured hemoglobin oxygen saturation and RBC tissue fraction continuously during the tests with an acquisition rate of 1 Hz. The MSI and EPOS systems used separate computers with synchronized clocks allowing for comparison of timestamps of the collected data. The room was dark, except from the LED light source, which was turned on during data acquisition. Dark calibration data, with the LED light source turned off, were acquired for three 10-s periods at t=4, 9, and 19 min after measurement start for both occlusion protocols to account for potential drift in the dark spectrum during the measurement. The dark calibration data-points were excluded before analysis of the measurements. EPOS measured hemoglobin oxygen saturation and RBC tissue fraction continuously during the tests.

When protocols were finished, white reference data were acquired to account for possible spectral changes in camera and light source over time. A white reference, Spectralon® tile (Labsphere, Inc., New Hampshire), was placed in the field of view of the MSI camera, at the same distance as the forearm surface. Data were acquired with the light source turned on and off for 10 s, respectively. Integration time was halved compared to measurements on tissue due to high reflectivity of the Spectralon® tile.

### 2.5 Data Pre-Processing

Each 2048×1088  pixel image captured by the MSI camera was spectrally sorted by the 16 wavelength bands/channels, n, resulting in a raw data multispectral data cube, $I^{M}(x,y,n)$, with 16 images of size 512×272  pixels. Note that $I^{M}(x,y,n)$ varies with time. The black ink dots on the arm were used to mark and track the analyzed skin location throughout the duration of the protocol. In the first image of each measurement sequence, the approximate location of the dots were marked manually. The exact location for each recorded image was then found by masking an area of 16×16  pixels around the dot and finding the “centre of mass” in an inverted and bandpass filtered image. The filtering mainly removed the average image intensity and finer objects such as hairs, leaving the position of the dots unaffected. The found coordinates were used as candidates for dot coordinates in the consecutive image and this was repeated for all measured samples. Afterward, all coordinates were examined manually without finding any false detection.

The measured dark calibration data cube, $I_{d}^{M}(x,y,n),$ was subtracted from each acquired data cube. $I_{d}^{M}(x,y,n)$ was a linear interpolation based on all three dark measurements acquired at t=4, 9, and 19 min.

White normalization data were dark corrected by $I_{d,w}^{M}(x,y,n)$, measured on the Spectralon® tile with the light source turned off. Since the tile was too small to cover the whole field of view, white normalization data from all included subjects were averaged to one common white normalization data cube, $I_{w}^{M}(x,y,n)$. The white normalized and dark calibrated data cube derived from measured data, $I_{N}^{M}(x,y,n),$ was calculated using:

$$I_{N}^{M}(x,y,n)=\frac{I^{M}(x,y,n)-I_{d}^{M}(x,y,n)}{I_{w}^{M}(x,y,n)-I_{d,w}^{M}(x,y,n)}.$$ (1)

An average intensity spectrum, $I_{N}^{M}{\left(x,y,n\right)}_{x,y}=I_{N}^{M}(n)$, was calculated over the 2×2  cm area of interest defined by the ink dots. The four MSI camera channels with main sensitivity peaks below 500 nm were excluded, due to low light source intensity for those wavelengths, and hence a high level of noise. The resulting spectrum was used in the inverse Monte Carlo analysis for temporal estimation of hemoglobin oxygen saturation and RBC tissue fraction, described in Sec. 2.8.

### 2.6 Tissue Model and Monte Carlo Simulations

The algorithm used for analyzing MSI data is based on a look-up table with pre-simulated Monte Carlo data from photon transport in a two-layered skin model.^20^ It is a simplified model of real skin tissue with homogeneous layers and thus provides no representation of blood vessels or other structures. Absorbers other than melanin and hemoglobin are also disregarded. The model is defined by reduced scattering coefficient $\mu_{s}^{'}(\lambda )$, melanin absorption coefficient $\mu_{a,\mathrm{mel}}(\lambda )$, and blood absorption coefficient $\mu_{a,b}(\lambda )$. The coefficients are determined by six parameters: tissue scattering (parameters α and β), tissue fraction melanin ($f_{\mathrm{mel}}$), RBC tissue fraction ($f_{\mathrm{RBC}}$), hemoglobin oxygen saturation ($S_{O_{2}}$), and epidermal thickness ($T_{\mathrm{epi}}$) in accordance with Eqs. (25).^21^ In our earlier study,^22^ we found that varying only $f_{\mathrm{mel}}$, $f_{\mathrm{RBC}}$, and $S_{O_{2}}$ during optimization is enough. Therefore, parameters α, β, and $T_{\mathrm{epi}}$ were set to fixed values, as described below.

$\mu_{s}^{\prime }(\lambda )$, given by Eq. (2),^21^ was assumed to be the same for both modeled layers. Parameters α and β were fixed to $\alpha =2.5\;{\mathrm{mm}}^{-1}$ and β=2, which is comparable to mean values from individual $\mu_{s}^{\prime }(\lambda )$, estimated by the EPOS system in this and other studies.^23^ $\mu_{s}(\lambda )$ is described by the Henyey–Greenstein scattering phase function in Eq. (3),^21^ with anisotropy factor g=0.8 for all wavelengths   λ.

$$\mu_{s}^{\prime }(\lambda )=\alpha \cdot {\left(\frac{\lambda }{500}\right)}^{-\beta },$$ (2)

$$\mu_{s}(\lambda )=\frac{\mu_{s}^{\prime }(\lambda )}{1-g}.$$ (3)

The absorption coefficient was different in the two layers. The upper epidermal layer had a thickness $T_{\mathrm{epi}}=125\;\mu m$ and contained melanin but no blood. Melanin absorption coefficient was given by Eq. (4),^21^ where $c_{\mathrm{mel}}$ was allowed to range from 0 to 0.084.

$$\mu_{a,\mathrm{mel}}(\lambda )=f_{\mathrm{mel}}\cdot 51.9\cdot {\left(\frac{\lambda }{500}\right)}^{-3}.$$ (4)

The lower dermal layer was considered infinitely thick and contained blood but no melanin. Blood absorption coefficient was given by Eq. (5),^21^ where $S_{O_{2}}\;$ranges from 0 (desaturated) to 1 (fully saturated), $f_{\mathrm{RBC}}$ ranges from 0 to 0.056, $\mu_{a,\mathrm{Hb}}$is the absorption coefficient of deoxygenated hemoglobin, and $\mu_{a,{\mathrm{HbO}}_{2}}$ is the absorption coefficient of oxygenated hemoglobin. The latter two were given by combining tabulated data from Zijlstra and Prahl, as described by Fredriksson et al.^18^

$$\mu_{a,b}(\lambda )=f_{\mathrm{RBC}}\cdot 0.15\cdot [(1-S_{O_{2}})\cdot \mu_{a,\mathrm{Hb}}(\lambda )+S_{O_{2}}\cdot \mu_{a,{\mathrm{HbO}}_{2}}(\lambda )].$$ (5)

Monte Carlo simulations were performed for chosen $\mu_{s}^{\prime }$ and$\;T_{\mathrm{epi}}$ until 200,000 photons were detected. Each photon’s path length in each layer was registered and subsequently used for adding absorption in accordance with Beer–Lambert’s law (i.e., white Monte Carlo). Resulting intensities were saved in a look-up table $H_{\mathrm{LUT}}^{\mathrm{MC}}(\mu_{s}^{\prime },\mu_{a,\mathrm{mel}},\mu_{a,b},T_{\mathrm{epi}})$. Interpolation within the dataset allows for fast generation of simulated diffuse reflectance spectra, $H^{\mathrm{MC}}(\lambda )$, based on simulated data with scattering and absorption properties varied within specified limits.

### 2.7 Post-processing of Monte Carlo Simulated Spectra

**Fig. 1:**
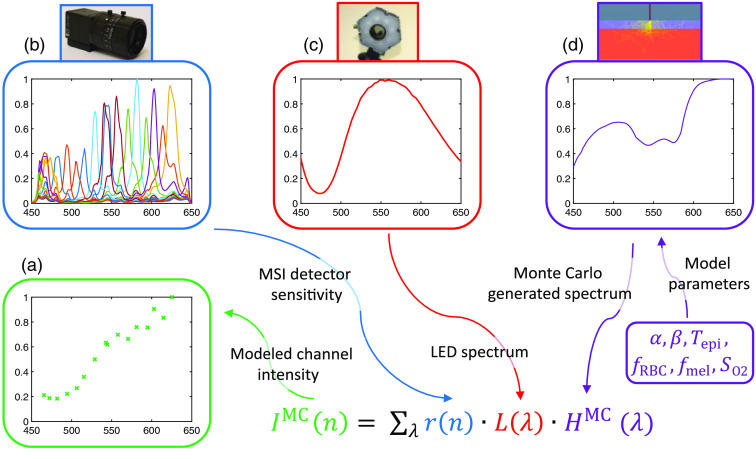
An outline describing how the modeled intensities for the 16 channels (a) are calculated. For each channel, the modeled intensity is found by summarizing the channel detector sensitivity (b), the light source emission spectrum (c), and the Monte Carlo generated spectrum (d) over the wavelength range as described by Eq. (6). The Monte Carlo generated spectrum is found in the pre-simulated look-up table for a combination of model parameters.

Post-processing of Monte Carlo simulated spectra is needed due to the substantial spectral overlap of the 16 wavelength channels of the MSI camera. This was previously described in Ewerlöf et al.^15^ To compare a Monte Carlo generated spectrum, $H^{\mathrm{MC}}(\lambda )$, with the measured 16 channel spectrum, the intensity, $I^{\mathrm{MC}}(n)$, corresponding to each channel, was calculated from Eq. (6), where $r_{n}(\lambda )$ is the camera detector sensitivity given by the manufacturer for each channel and L(λ) is the output spectrum of the light source. The response is summarized for all wavelengths in the range of 450 to 650 nm to get the total intensity response from each channel. The procedure is illustrated in Fig. 1.

$$I^{\mathrm{MC}}(n)=\;\underset{\lambda }{\sum }r_{n}(\lambda )\cdot L(\lambda )\cdot H^{\mathrm{MC}}(\lambda ).$$ (6)

The corresponding white normalization,$I_{w}^{\mathrm{MC}}(n)$, was calculated by Eq. (7), using the generated spectrum $H_{w}^{\mathrm{MC}}(\lambda )=1$, and then used in Eq. (8) to get the white normalization intensity response, $I_{N}^{\mathrm{MC}}(n)$, for a Monte Carlo generated spectrum.

$$I_{w}^{\mathrm{MC}}(n)=\underset{\lambda }{\sum }r_{n}(\lambda )\cdot L(\lambda )\cdot 1,$$ (7)

$$I_{N}^{\mathrm{MC}}(n)=\frac{I^{\mathrm{MC}}(n)}{I_{w}^{\mathrm{MC}}(n)}.$$ (8)

### 2.8 Inverse Monte Carlo Algorithm

The inverse Monte Carlo algorithm was previously described in Refs. 15 and 22. Each measured intensity response from the MSI camera, $I_{N}^{M}(n)$, is compared to Monte Carlo generated data, $I_{N}^{\mathrm{MC}}(n)$, modeled with known optical properties. The penalty function, E [Eq. (9)], is constructed from two parts described by Eqs. (10) and (11).

E=[EI(n)10·ES]. (9)

Monte Carlo generated spectra, $I_{N}^{\mathrm{MC}}(n)$, are compared to the measured spectrum, $I_{N}^{M}(n)$, using Eq. (10).

$$E_{I}(n)=\;\frac{I_{N}^{\mathrm{MC}}(n)}{I_{N}^{\mathrm{MC}}(n{)}_{n}}/\frac{I_{N}^{M}(n)}{I_{N}^{M}{\left(n\right)}_{n}}-1.\;$$ (10)

To further improve sensitivity to hemoglobin oxygen saturation, four wavelength channels were chosen for their high sensitivity in the wavelength region of 540 to 580 nm, where oxy- and deoxyhemoglobin absorption spectra have distinct differences. The ratio between channels was calculated and multiplied by 10 in the penalty function, to give the term high impact on the calculated error. Numbers in parenthesis in Eq. (11) denote the wavelength of the main peak of the detector sensitivity channel.

$$E_{S}=\frac{I_{N}^{\mathrm{MC}}(557\;\mathrm{nm})+I_{N}^{\mathrm{MC}}(570\;\mathrm{nm})}{I_{N}^{\mathrm{MC}}(544\;\mathrm{nm})+I_{N}^{\mathrm{MC}}(580\;\mathrm{nm})}/\frac{I_{N}^{M}(557\;\mathrm{nm})+I_{N}^{M}(570\;\mathrm{nm})}{I_{N}^{M}(544\;\mathrm{nm})+I_{N}^{M}(580\;\mathrm{nm})}-1.$$ (11)

The best-fit is found by minimizing the penalty function, E, in Eq. (9) in a square law sense with a trust-region-reflective algorithm described by Eq. (12).

$$\underset{S_{O_{2}},\;f_{\mathrm{RBC}},\;f_{\mathrm{mel}}}{\min}{∥E∥}_{2}^{2}.$$ (12)

### 2.9 Statistics

Linear regression analysis and Bland–Altman analysis were performed on the results. All measurement values from the MSI camera and EPOS were synced in time by comparing absolute timestamps from each respective system and interpolating EPOS data to match the timestamps of MSI data.

Hemoglobin oxygen saturation values from MSI and EPOS are easily compared since both are measured in %. Measurements from a time interval of 9 min (1 min before occlusion until 3 min after release) were compared using linear regression analysis. The time interval was chosen to level out the distribution of saturation values between high and low values. Saturation values are generally stable during baseline and return to baseline values a few minutes after release, which leads to an overrepresentation of values in this range if all measurements were to be included.

The Bland–Altman method^24^ was used to compare measurement methods MSI and EPOS. The analysis was done for three time intervals: baseline, end of occlusion, and soon after release.^25^ To avoid measurements during inflation of the occlusion cuff, values during 55 s of baseline measurements (from 60 s before until 5 s before occlusion starts) were used for the baseline analysis. The end of occlusion interval (from 60 s before until 10 s before release) was chosen accordingly. After release, reperfusion is distinct. To capture the maximum saturation in the Bland–Altman analysis, we chose to use the median of nine samples around the maximum value in the interval from 5 s before release until 55 s after release. The mean value was calculated for each time interval for MSI and EPOS measurements, respectively. Mean bias was calculated along with upper and lower limits set to mean±1.96 standard deviations.

It is not possible to compare RBC tissue fraction values between MSI (relative units) and EPOS (% tissue fraction), without scaling MSI data. Therefore, a mean RBC tissue fraction value, $\left\langle f_{\mathrm{RBC}}\right\rangle$, assessed from data averaged in the interval 1 min before occlusion until 3 min after release, including all subjects, was calculated for MSI and EPOS data separately. The MSI data were then calibrated by multiplication with $\left\langle f_{\mathrm{RBC},\;\mathrm{EPOS}}\rangle /\langle f_{\mathrm{RBC},\mathrm{MSI}}\right\rangle$. Linear regression analysis was used to compare the results. Bland–Altman analysis was also performed as described above for data captured at baseline and during occlusion. RBC tissue fraction values acquired during the reperfusion phase do not differentiate from levels found during baseline and occlusion and are hence disregarded.

To find possible differences in sensitivity to RBC tissue fraction between the two methods, ratios of RBC tissue fraction values at the end of occlusion and during baseline were calculated for both arterial and venous occlusion. The standard deviation for the ratio was higher for MSI than for EPOS, particularly during arterial occlusion (Table 2). Therefore, the ratio from MSI was compared to that from EPOS using the non-parametric Wilcoxon signed-rank test.

### 2.10 Penetration Depth for MSI and EPOS

A set of Monte Carlo simulations were conducted for verification of sampling depth for the MSI and EPOS systems. Tissue mimicking values for $\mu_{s}^{\prime }(\lambda )$ and $\mu_{a}(\lambda )$ were used at wavelengths 475 and 630 nm. The tissue optical properties, compiled from an *in-vivo* population-based study^23^ [$\mu_{a}(\lambda )$ values are preliminary and not published yet] were 3.20 and 1.80 (${\mathrm{mm}}^{-1}$) for $\mu_{s}^{\prime }(\lambda )$ and 0.20 and 0.01 (${\mathrm{mm}}^{-1}$) for $\mu_{a}(\lambda )$, respectively.

### 2.11 Data Exclusion

Data from three subjects were excluded due to a poor EPOS signal (one subject) and exclusion criteria (one subject had coffee prior to the measurements, and one had a skin condition). One additional subject was excluded from the venous occlusion protocol due to non-adherence to study protocol. The remaining data used for the statistical analysis were collected from 21 subjects during arterial occlusion and 20 subjects during venous occlusion.

## 3 Results

**Fig. 2:**
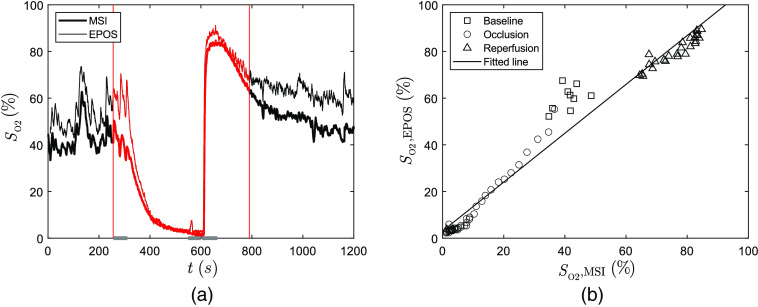
(a) Hemoglobin oxygen saturation ($S_{O_{2}}$) during a typical arterial occlusion (MSI = bold line, EPOS = thin line). Occlusion starts at t=300  s and ends at t=600  s. Vertical lines indicate time interval used for regression analysis (240 to 780 s). Time intervals used for Bland–Altman analysis are marked in gray for baseline (240 to 295 s), occlusion (540 to 590 s), and reperfusion (595 to 655 s). (b) Linear regression analysis of data in (a) (every 10th data point). Data during baseline (squares), occlusion (circles), reperfusion (triangles), and the fitted regression (solid line) are presented.

Table 1 presents the average hemoglobin oxygen saturation and RBC tissue fraction estimated by MSI and EPOS systems during arterial and venous occlusion of all subjects during the three time intervals indicated by gray horizontal lines in Fig. 2(a). The subject used as an example in Figs. 2, 4, and 5 was chosen based on the correlation coefficient $R^{2}$ between MSI and EPOS estimated values of hemoglobin oxygen saturation being closest to the mean of all subjects.

**Table 1:** Hemoglobin oxygen saturation ($S_{O_{2}}$ ) and RBC tissue fraction ($f_{\mathrm{RBC}}$) estimated by MSI and EPOS at baseline, end of occlusion, and start of reperfusion (mean (SD) and [min max] for all included subjects). For $f_{\mathrm{RBC}}$, the second line presents MSI values calibrated to EPOS data.

		Baseline	End of occlusion	Reperfusion
$S_{O_{2}}\;$during arterial occlusion	MSI (%)	42.6 (9.9)	1.5 (2.4)	82.4 (7.4)
		[21.7 62.7]	[0 9.5]	[55.6 93.8]
	EPOS (%)	55.8 (10.3)	1.8 (1.7)	88.2 (5.7)
		[39.5 71.7]	[−0.6 5.5]	[69.2 96.2]
$f_{\mathrm{RBC}}$ during venous occlusion	MSI (-)	0.012 (0.0042)	0.044 (0.0093)	0.025 (0.0064)
		[0.0050 0.021]	[0.025 0.062]	[0.013 0.034]
	Calibrated MSI (%)	0.28 (0.095)	1.00 (0.21)	0.56 (0.15)
		[0.12 0.48]	[0.58 1.42]	[0.29 0.77]
	EPOS (%)	0.46 (0.12)	0.86 (0.18)	0.57 (0.13)
		[0.18 0.71]	[0.50 1.21]	[0.32 0.80]

The changes in oxygen saturation for the chosen subject during an arterial occlusion provocation are presented in Fig. 2(a). The mean oxygen saturation level at baseline estimated by the MSI system is 41.5% in the first indicated time interval (gray horizontal lines). When occlusion is initiated, it decreases and after 5 min of occlusion, oxygen saturation levels are down to only 2%. When occlusion is released, oxygen saturation peaks at 84.7% during the hyperemic period. Oxygen saturation then decreases toward the baseline level.

Oxygen saturation estimated by the MSI system was compared to EPOS data by linear regression analysis. Figure 2(b) presents sampled data from Fig. 2(a) and the plotted linear regression. For this subject, $R^{2}$ is 0.964 and the slope of the line is 1.05. For the whole population of 21 included subjects, the mean value of $R^{2}$ is 0.958 with values ranging between 0.904 and 0.993.

**Fig. 3:**
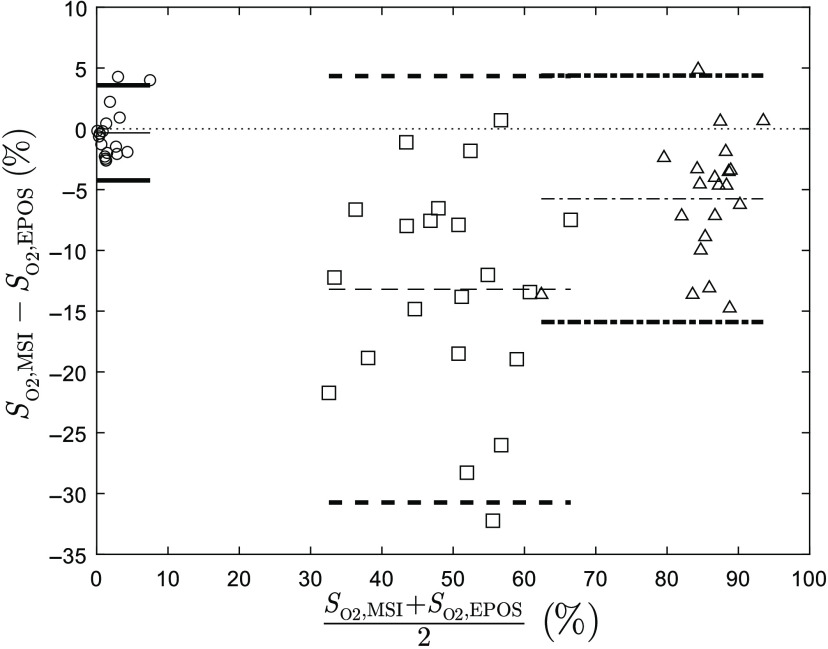
Agreement of hemoglobin oxygen saturation $\left(S_{O_{2}}\right)$ between values estimated by MSI and EPOS from Bland–Altman analysis of 21 subjects at three separate time intervals [see Fig. 2(a)] during arterial occlusion: baseline (squares/dashed), end of occlusion (circles/solid lines), and reperfusion (triangles/dash-dotted lines). The mean difference for each phase is represented by thin lines and the difference ±1.96 * standard deviation (95% prediction interval) by bold lines.

Bland–Altman analysis was performed for 21 subjects at three chosen time intervals indicated in Fig. 2(a). The result is presented in Fig. 3. MSI and EPOS methods give similar results for low oxygen saturation at the end of occlusion. The mean bias is 0.3% with upper and lower limits of 3.6% and −4.2% deviation, respectively. Baseline measurements show a larger difference with a mean bias of 13.2% with upper and lower limits of 4.3% and −30.7% deviation. EPOS values are generally considerably higher than MSI values. Higher EPOS values are also found during reperfusion but not to the same extent. Mean bias is 5.6% with upper and lower limits of 4.4% and −15.9% deviation, respectively.

**Fig. 4:**
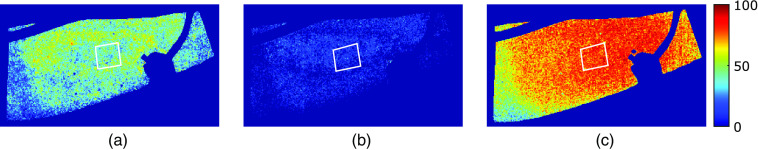
Spatially resolved oxygen saturation estimated by MSI. For baseline (a) and occlusion (b), the last timepoints of the intervals indicated in Fig. 2(a) were selected, whereas the timepoint with the peak oxygen saturation in the interval was selected for reperfusion (c). The ROI used for averaging data presented in Fig. 2 is outlined by the white square.

Figure 4 shows spatially resolved oxygen saturation images calculated at three different temporal points for the same subject as in Fig. 2. At baseline and occlusion, the last timepoint in the intervals indicated in Fig. 2(a) was selected, whereas the timepoint with the peak oxygen saturation was selected within the reperfusion interval. The three intensity data cubes acquired by the MSI system were analyzed pixel by pixel, displaying the oxygen saturation of the lower arm.

**Fig. 5:**
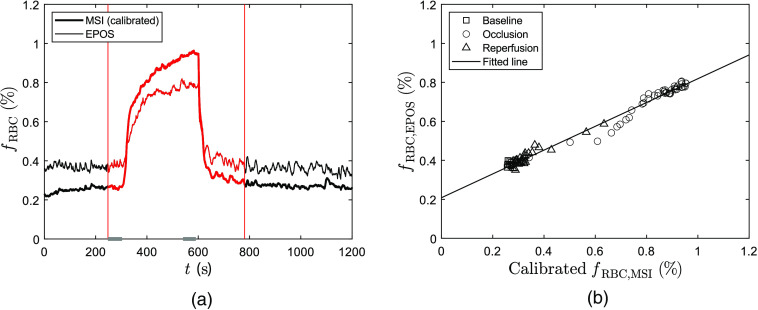
(a) RBC tissue fraction ($f_{\mathrm{RBC}}$) during a typical venous occlusion (calibrated MSI = bold line, EPOS = thin line). For MSI data calibration, see Sec. 2.9. Occlusion starts at t=300  s and ends at t=600  s. Vertical lines indicate time interval used for regression analysis (240 to 780 s). Time intervals used for Bland–Altman analysis are marked in gray for baseline (240 to 295 s) and occlusion (540 to 590 s). (b) Linear regression analysis of data in (a) (every 10th data point). Data during baseline (squares), occlusion (circles), reperfusion (triangles), and the fitted regression (solid line) are presented.

RBC tissue fraction data estimated during venous occlusion for the same subject as in Fig. 2 are presented in Fig. 5(a). Levels are stable during baseline but increase rapidly when the arm is occluded. During occlusion, the increase continues but at a slower rate. Right after occlusion release, levels drop and soon reach baseline level again. Estimated EPOS RBC tissue fraction values are 0.38% at baseline and 0.79% at the end of occlusion. MSI data are calibrated as described in Sec. 2.9 prior to comparison with EPOS data.

Linear regression analysis was performed on RBC tissue fraction values at venous occlusion for all 20 included subjects. Figure 5(b) presents regression analysis of data in Fig. 5(a). For this subject, $R^{2}$ is 0.982 and the slope of the line is 0.61. For all included subjects, the mean value of $R^{2}$ is 0.925 with values ranging between 0.717 and 0.984. $R^{2}>0.9$ for 75% of the subjects.

**Fig. 6:**
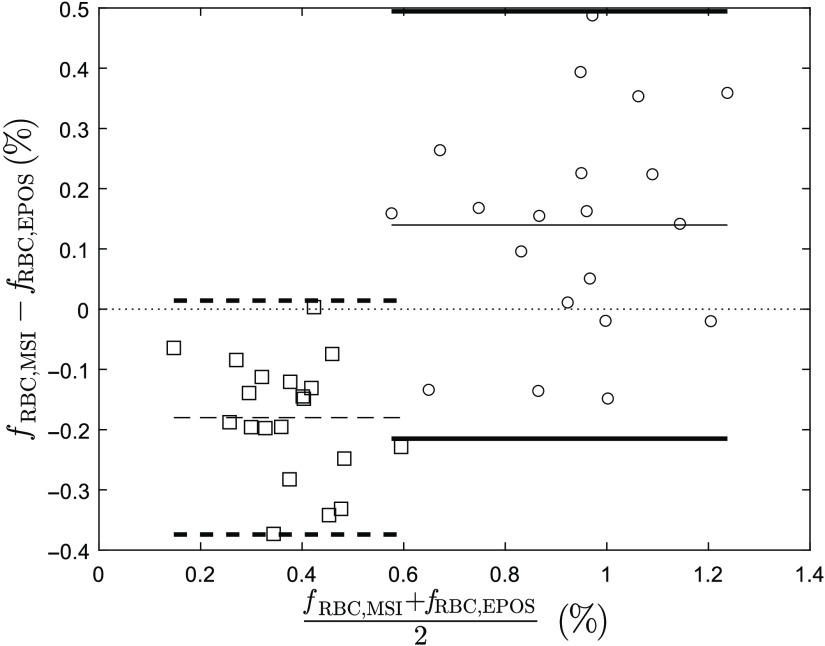
Agreement of RBC tissue fraction ($f_{\mathrm{RBC}}$) between values estimated by MSI (calibrated) and EPOS from Bland–Altman analysis of 20 subjects at two separate time intervals [see Fig. 5 (a)] during venous occlusion: baseline (squares/dashed lines) and during end of occlusion (circles/solid lines). The mean difference for each phase is represented by thin lines and the difference ±1.96 * standard deviation (95% prediction interval) by bold lines.

Results from Bland–Altman analysis of 20 subjects are presented in Fig. 6 for two time intervals indicated in Fig. 5(a). After normalization, RBC tissue fraction is underestimated by MSI measurements at baseline with mean bias −0.18 and upper and lower limits 0.014 and −0.37, respectively, compared with EPOS. At the end of venous occlusion, RBC tissue fraction is underestimated by MSI compared to EPOS with mean bias 0.14 and upper and lower limits 0.49 and −0.21, respectively.

The ratios of estimated RBC tissue fraction between end of occlusion and baseline, by MSI and EPOS, are presented in Table 2 for both arterial and venous occlusion. The ratio was significantly larger when measuring with MSI (p<0.001, arterial and venous occlusion), and most distinct during venous occlusion when change in RBC tissue fraction is apparent.

Estimated average maximal photon penetration depth at the two wavelengths 475 and 630 nm is 0.43 and 0.79 mm with EPOS (each value is an average of the result for the DRS source detector fiber at 0.40 and 1.20 mm distances), respectively. Similar MSI values are 0.45 and 1.79 mm, respectively.

**Table 2:** Ratio of estimated RBC tissue fraction between end occlusion and at baseline for MSI and EPOS during arterial and venous occlusion (mean and (SD) for all included subjects).

	MSI	EPOS
Arterial occlusion	2.24 (0.83)	1.26 (0.23)*
Venous occlusion	3.77 (1.04)	1.93 (0.41)*

* p<0.001 EPOS versus MSI.

## 4 Discussion

In this study, we have advanced and evaluated an algorithm estimating microvascular hemoglobin oxygen saturation and RBC tissue fraction from collected snapshot MSI data. The algorithm, based on inverse Monte Carlo modelling, uses six parameters to model light transport in tissue, with three of the parameters assigned fixed values and three varying within a specified range relevant to skin tissue. Measurements during venous and arterial forearm occlusions of 24 subjects show that assessment of hemoglobin oxygen saturation and RBC tissue fraction with our MSI system and algorithm is highly correlated with those measurements using a probe-based reference system. However, there are systematic differences that might be explained by differences in sampling volume.

When synchronizing in time between MSI and EPOS, reactions to occlusion and reperfusion seem to be concurrent in both methods. Brachial vascular occlusion affects the whole arm distally, and therefore the two measurement sites (MSI and EPOS) are influenced similarly. Spatial differences in tissue compounds between the sites, for example, presence of shallow or large vessels or other structures, may impact the result at an individual level but are averaged out within a population.

Hemoglobin oxygen saturation values are estimated and verified through a linear regression analysis showing a mean correlation coefficient $R^{2}=0.956$, compared to the EPOS reference system. Applying Bland–Altman analysis of MSI and EPOS shows that agreement is best at the end of occlusion when oxygenation is low. There is an offset between MSI and EPOS measurements during baseline, where MSI shows a lower hemoglobin oxygen saturation.

Since MSI RBC tissue fraction values are relative in contrast to values measured by EPOS, MSI values are calibrated to those from EPOS before comparison. Applying linear regression analysis shows an $R^{2}=0.927$, whereas the Bland–Altman analysis reveals that MSI underestimates at baseline and similarly overestimates the end of occlusion values compared to EPOS RBC tissue fraction results.

The additional Monte Carlo simulation on penetration depth shows that, for longer wavelengths where light absorption of hemoglobin is low, the MSI system has a sampling volume reaching substantially deeper into tissue (1.79 mm) compared to EPOS (0.79 mm). The most superficial veins are found at a depth around 0.75 mm in forearm tissue,^26^ which suggests that they are mainly undetectable to the EPOS system. The systematic differences between the MSI and EPOS values may be explained by this difference in sampling volumes, effectively making the MSI measurements more influenced by venous blood.

During end of arterial occlusion, both MSI and EPOS sample a vascular bed with essentially deoxygenated hemoglobin. Similarly, both methods sample a vascular bed with essentially oxygenated blood soon after release of the occlusion due to a short capillary transit time and a low oxygen extraction during hyperemia, caused by a high blood flow speed. However, at baseline, the difference in oxygenation between vascular plexa is more pronounced, which makes the different sampling volumes of the two modalities affect their oxygen measures more strongly.

Sampling volume differences can also explain the larger change in RBC tissue fraction measured by MSI than EPOS when comparing baseline and end of occlusion data in Table 2. The observation holds both for arterial and venous occlusion but is more expressed for the latter. Deeper lying venous vessels have a high capacitance and the RBC tissue fraction in these vessels is increased when blood is accumulated during occlusion. The MSI with its deeper sampling volume will sample more of the venous blood than EPOS, which would explain the difference in estimation of the blood volume.

During venous occlusion, RBC tissue fraction increases gradually throughout the length of the occlusion period as expected. When analyzing RBC tissue fraction levels during arterial occlusion (data not presented), RBC tissue fraction similarly increases during cuff inflation but also changes after the cuff pressure reach levels well above the arterial pressure. This could be explained by a redistribution of blood driven by the initial pressure difference between arteries and veins, causing blood to flow from deep arteries positioned well below our sampling depth to veins located deep but visible to our measurement systems.

It is possible to apply the described algorithm on individual pixels of the MSI data cube to produce spatially resolved results, as illustrated in Fig. 4. In this study, MSI data from a 2×2  cm large area was averaged, analyzed, and compared to data from the point-wise measuring EPOS probe. The example in Fig. 4 shows small spatial variations in oxygen saturation within the ROI, which indicates that averaging has a limited impact on the result. The spatially averaged MSI data in comparison to the EPOS data may show less conformance due to different sampling sites. However, for the whole study, MSI and EPOS values agreed well, especially for hemoglobin oxygen saturation where the $R^{2}$-value is >0.90 for all subjects.

Other groups have used the snapshot MSI camera from XIMEA to measure skin tissue oxygen saturation. Bauer et al.^12^ describe a method for spectral correction of the XIMEA camera characteristics, where unwanted spectral peak sensitivity is decreased. This is essential when using inverse algorithms that do not fully account for the complete spectral sensitivity of each pixel, but instead analyze the data based on scattering and absorption values for a single wavelength (i.e., the peak wavelength). Our approach does not suffer from this limitation as the complete spectral behavior of both skin tissue and detector characteristics are accounted for. However, this is done at the expense of a computationally demanding algorithm based on Monte Carlo simulations, making it unsuitable for real-time analysis of complete images. Bauer et al. applied their algorithm to estimate relative changes in blood oxygen saturation on the back of the hand, using only 3 out of 16 available channels. The relatively low acquisition speed (1 fps) allowed values to be calculated in real time. To evaluate the system dynamically, they used arterial occlusion of the upper arm. The results are compared with a standardized probe-based system that measures in the near-infrared (NIR) regime^27^ and sampling volume for the two systems differ considerably.^12^ Bauer et al. found differences in oxygenation during occlusion like we did, but it is not possible to compare estimated oxygen saturation values, as all presented values are normalized to the range [0 1] and compare only shapes of the curves.

Rubins et al. used a k-means segmentation algorithm and a two-layered optical diffuse model to relate intensities measured from palm side of the hand by the XIMEA camera to skin oxygen saturation levels. Their protocol involved upper arm occlusion to 150 mmHg for 10 min.^13^ The results cannot be compared to our values as values are not quantitative or compared to a reference method. The cuff pressure was considerably lower than the pressure used in our protocol and might not have given a similar response. Furthermore, the dynamic response in the reperfusion phase was limited to the same level as oxygen saturation values estimated during baseline (100%).^13^

The sensitivity to the hemoglobin absorption spectra is influenced by key wavelengths and an analysis algorithm may be improved by a wise selection or combination of channels.^22^^,^^28^ However, these sensors are unique, and spectral properties, such as peak wavelength, vary between specimens. Therefore, when designing algorithms that are expected to work not only for a single camera, fixed pre-selected wavelengths or channels are not a viable option. In this work, we present an optional strategy that addresses this. In general, our approach appears to work well. Some residuals are, however, present in our non-linear optimization algorithm when fitting simulated data to measured data, which might explain some of the differences found between the two compared modalities. The origin of these residuals can most likely be found in the spectral sensitivity provided by the manufacturer. Preliminary data indicate that these spectral sensitivities might not be exact when adding an optical lens to the sensor, which calls for further calibration measurements.

## 5 Conclusion

We have demonstrated that an inverse Monte Carlo algorithm, based on a two-layer skin model with three free parameters, can accurately and consistently model spectra captured using an MSI snapshot camera with complex filter characteristics. Data acquired from forearm skin during arterial and venous occlusion show that the hemoglobin oxygen saturation and RBC tissue fraction estimated from MSI data have a high correlation with values co-registered with the validated probe-based EPOS system. Some systematic differences were found, but these may be explained by a larger sampling volume for the MSI system at longer wavelengths where tissue absorption is low.
